# Novel multivalent S100A8 inhibitory peptides attenuate tumor progression and metastasis by inhibiting the TLR4-dependent pathway

**DOI:** 10.1038/s41417-023-00604-3

**Published:** 2023-03-17

**Authors:** Atsuko Deguchi, Miho Watanabe-Takahashi, Taishi Mishima, Tsutomu Omori, Umeharu Ohto, Nobuto Arashiki, Fumio Nakamura, Kiyotaka Nishikawa, Yoshiro Maru

**Affiliations:** 1 Department of Pharmacology, Tokyo Women’s Medical University, 8-1 Kawada-cho, Shinjuku-ku, Tokyo, 162-8666 Japan; 2 Faculty of Life and Medical Sciences, Doshisha University, 1-3 Miyakotani, Tatara, Kyotanabe, Kyoto, 610-0321 Japan; 3 Graduate School of Pharmaceutical Sciences, The University of Tokyo, Hongo, Bunkyo-ku, Tokyo, 113-0033 Japan; 4 Department of Biochemistry, Tokyo Women’s Medical University, 8-1 Kawada-cho, Shinjuku-ku, Tokyo, 162-8666 Japan

**Keywords:** Drug development, Cancer microenvironment

## Abstract

The tumor-elicited inflammation is closely related to tumor microenvironment during tumor progression. *S100A8*, an endogenous ligand of Toll-like receptor 4 (TLR4), is known as a key molecule in the tumor microenvironment and premetastatic niche formation. We firstly generated a novel multivalent S100A8 competitive inhibitory peptide (divalent peptide3A5) against TLR4/MD-2, using the alanine scanning. Divalent peptide3A5 suppressed S100A8-mediated interleukin-8 and vascular endothelial growth factor production in human colorectal tumor SW480 cells. Using SW480-transplanted xenograft models, divalent peptide3A5 suppressed tumor progression in a dose-dependent manner. We demonstrated that combination therapy with divalent peptide3A5 and bevacizumab synergistically suppressed tumor growth in SW480 xenograft models. Using syngeneic mouse models, we found that divalent peptide3A5 improved the efficacy of anti-programmed death (PD)1 antibody, and lung metastasis. In addition, by using multivalent peptide library screening based on peptide3A5, we then isolated two more candidates; divalent ILVIK, and tetravalent ILVIK. Of note, multivalent ILVIK, but not monovalent ILVIK showed competitive inhibitory activity against TLR4/MD-2 complex, and anti-tumoral activity in SW480 xenograft models. As most tumor cells including SW480 cells also express TLR4, S100A8 inhibitory peptides would target both the tumor microenvironment and tumor cells. Thus, multivalent S100A8 inhibitory peptides would provide new pharmaceutical options for aggressive cancers.

## Introduction

The formation of a premetastatic niche has been widely accepted as an indicator of metastasis. Similar to tumor microenvironment formation, various types of cells, such as immune cells, endothelial cells, and fibroblasts, are involved in premetastatic niche formation. We have discovered that primary tumors hijack Toll-like receptor 4 (TLR4) signaling to establish a premetastatic niche in the lung by utilizing endogenous ligands [1].

Metastasis is a continuous and multi-step biological process, in which a small population of cancer cells with highly invasive and metastatic potential secedes from its primary tumor. During metastasis, tumor cells digest the extracellular matrix, intravasate into the blood or lymphatic vessels, and extravasate to and colonize distant organs. The theory of “Seed and Soil” proposed by Paget [2] has been widely accepted. To date, tumor-derived secreted cytokines, such as vascular endothelial growth factor (VEGF), tumor necrosis factor (TNF), stromal cell-derived factor 1, and transforming growth factor-β (TGF-β), which form premetastatic niches, induce the mobilization of bone marrow-derived cells. The recruitment of vascular endothelial growth factor receptor 1 hematopoietic bone marrow progenitors, CD11b^+^ myeloid cells, and suppressive immune cells initiates the pre-metastatic niches, thereby promoting metastasis [3]. We previously identified *S100A8* as one of the upregulated genes in the lungs of tumor-bearing mice [4]. Similar to S100A9, S100A8 is an endogenous ligand of TLR4 [5].

Toll-like receptors (TLRs), the best-characterized family of pattern recognition receptors, have been considered for their ability to respond to exogenous pathogen-associated molecular patterns (PAMPs), including bacterial diacylated and triacylated lipopeptides, bacterial lipopolysaccharide (LPS), bacterial flagellin, bacterial and viral unmethylated CpG-containing DNA motifs, and viral single- or double-stranded RNA [6]. Among TLRs, TLR4 activation via LPS is essential for host defense against gram-negative bacteria. In addition to PAMPs, TLR4 recognizes danger-associated molecular patterns (DAMPs). To date, high-mobility group box 1 (HMGB1) [7], S100A8/S100A9 [4, 8], serum amyloid A3 (SAA3) [1, 9], heat shock protein (Hsp) 60 [10], Hsp70 [11], fetuin-A [12], defensin-β [13], fibrinogen [14], and fibronectin [15], as well as polysaccharides such as hyaluronan [16], heparan sulfate [17], biglycan [18], and decorin [19] have been reported as endogenous ligands of TLR4. TLR4 associates with the co-receptor MD-2, and LPS binding to MD-2 induces the dimerization of TLR4 [20]. Intriguingly, accumulating evidence suggests that DAMPs-mediated signaling can promote metastasis. Initially, we thought that S100A8 mainly plays a role in premetastatic niche formation, but it has been reported that various types of cancer cells also express TLR4 at the cell surface. To date, S100A8 expression or overexpression of TLR4 has been observed in various cancers.

S100A8 belongs to the S100s family and is characterized by two calcium-binding EF-hand motifs: a carboxyl-terminal EF-hand (high affinity to calcium), and an N-terminal EF-hand (low affinity to calcium) that are connected by a central hinge. Like most S100 proteins, S100A8 can form a heterodimer with S100A9 or can exist as a homodimer or monomer [21]. Vogl et al. originally reported that S100A8-mediated cytokine induction was mediated via TLR4 [5]. Moreover, we previously found that the carboxyl terminal region of mouse S100A8 had a high affinity for TLR4/MD-2, and that S100A8 induced inflammatory cytokines/chemokines in a TLR4-dependent manner [8].

Myeloid-derived suppressor cells (MDSCs) are immature myeloid cells derived from the bone marrow. They are recruited to primary tumor sites and distant organs, which contributes to various immune responses [22]. The recruitment of MDSCs is a crucial step in the formation of premetastatic niche. Immunosuppression is a major characteristic of MDSCs. In mice, MDSCs can be divided into at least two populations: granulocytic/polymorphonuclear MDSCs (PMN-MDSCs) and monocytic MDSCs (M-MDSCs). In addition, early MDSCs have been identified in humans. Classical myeloid cell activation is driven mainly by TLR activation. Both M-MDSCs and PMN-MDSCs expressed S100A8/S100A9. PMN-MDSCs in the premetastatic niches may contribute to immune escape, inducing matrix remodeling and promoting angiogenesis, which in turn promotes the colonization of tumor cells.

We previously reported that Eritoran, a TLR4 inhibitor, inhibited S100A8-mediated cell migration and tumor progression in tumor-bearing mice [8]. Accumulating evidence suggests that S100A8 targeting can be therapeutically useful in advanced cancers, however there is no such treatment for S100A8 targeting. To validate the possibility of S100A8 targeting against aggressive cancers, we have developed novel multivalent S100A8 competitive inhibitory peptides, and found that S100A8 competitive inhibitory peptides suppress both tumor progression and experimental tumor metastasis.

## Materials and methods

### Reagents

Anti-phospho-p38 (Cat No. 9211), anti-phospho-p65 (Cat No. 3033), anti-p38 (Cat No. 9212), anti-p65 (Cat No. 8242), anti-S100A8 (Cat No. 9212), anti-CD8 (Cat No. 98941), anti-Cleaved-Caspase-3 (Cat No. 9661) antibodies, were purchased from Cell Signaling Technology (Danvers, MA, USA). Anti-TLR4 antibody (Cat No. ab13556), anti-Ki-67 (Cat No. ab16667) were purchased from Abcam (Cambridge, UK). Anti-S100A8 antibody were generated in our laboratory as described previously [8]. The purity of recombinant proteins was above 98% as judged by SDS-PAGE analysis. Endotoxin levels of recombinant proteins determined by LAL test (WAKO Chemicals, Japan) were <0.02 EU/µg. SW480 cell line was purchased from ATCC (Manassas, VA, USA), LLC cell line was purchased from RIKEN BioResource Research Center (Ibaraki, Japan) and MC38 cell line was kindly provided by Dr. Nicolas P. Restifo (NIH, Bethesda, MD). All cell lines were checked every two months, and found to be mycoplasma free.

### Peptides and library screening

Monovalent, divalent, and tetravalent peptides were synthesized using N-α-Fmoc-protected amino acids and standard BOP/HOB coupling chemistry [23]. The synthesized peptides were validated using mass spectrometry analysis in an Autoflex II TOF/TOF system (Bruker Daltonics, Billerica, MA, USA).

### Screening of divalent peptides synthesized on a membrane

Spot synthesis of divalent peptides on a cellulose membrane was performed using a ResPep SL SPOT synthesizer (INTAVIS Bioanalytical Instruments AG, Koeln, Germany) as described previously [24]. Briefly, Fmoc-β-Ala-OH (Watanabe Chemical Industries, Hiroshima, Japan) was used in the first cycle, followed by amino hexanoic acid as a spacer. Fmoc-Lys(Fmoc)-OH (Watanabe Chemical Industries) was used for the next cycle to form two branches in the peptide chain for the subsequent synthesis of the various motifs. The synthesis of each peptide was confirmed by staining the membrane using bromophenol blue (1% in *N, N*′-dimethylformamide), which reacts with free amino residues. Free amino residues are produced only after the completion of all reactions and before deprotection of the side chain residues. After destaining, the membrane was used for the affinity assay with TLR4/MD-2 protein.

### ELISA assay

S100A8 recombinant protein was purified as previously described [8]. These proteins were immobilized on Maxisorp plates (Thermo Fisher Scientific, Waltham, MA, USA), followed by blocking with protein-free blocking buffer (Thermo Fisher Scientific). TLR4/MD-2 recombinant proteins [25] with various concentrations of candidate peptides were then added to the indicated S100A8-immobilized ELISA plates for 2 h. The plates were washed, incubated with anti-TLR4 antibody, and HRP-conjugated secondary antibody, followed by the addition of substrate. The reaction was stopped by addition a 2N H_2_SO_4_ solution. Absorbance OD_450_/OD_650_ was measured using a microplate reader (Thermo Fisher Scientific). Maxisorp plates were also used for peptide coating. After blocking with protein-free blocking buffer, the coated plates were washed, and TLR4/MD-2 recombinant protein (2 µg/ml) was added to each well. The plates were incubated with a TLR4 antibody followed by horseradish peroxidase (HRP)-labeled anti-rabbit IgG. For detection of IL8 and VEGF in culture supernatants, SW480 cells were pretreated with the indicated peptides, and followed by stimulation with S100A8 for 24 h. The amount of interleukin-8 (IL-8) or VEGF in the supernatants were analyzed by ELISA kits (R&D Systems, Mineapolis, MN) according to the manufacturer’s instructions.

### Western blotting

Cells were lysed in lysis buffer (50 mM Tris-HCl [pH 7.4], 150 mM NaCl, 5 mM Na_4_P_2_O_7_, 10 mM β-glycerophosphate, 25 mM NaF, 1 mM EDTA, 1 mM EGTA, 1 mM Na_3_VO_4_, and 1% [v/v] Triton X-100) containing the cOmplete^TM^ Mini protease inhibitor cocktail (Roche Diagnostics, Indianapolis, IN, USA) as described previously [8]. Cell lysates (20 µg) were separated using SDS-PAGE and transferred to an Immun-Blot PVDF membrane (Bio-Rad, Hercules, CA, USA). After blocking with Blocking One or Blocking One-P (Nacalai tesque, Kyoto, Japan), membranes were probed with the indicated antibodies, followed by HRP-labeled anti-rabbit IgG and anti-mouse IgG (GE Healthcare). Signals were visualized using Immobilon Western HRP Substrate (Millipore, Billerica, USA).

#### Quantitative PCR analysis

SW480 cells were treated with S100A8 in Dulbecco’s Modified Eagle Medium/HamF12 supplemented with 0.1% bovine serum albumin for cytokine induction. Total RNA was extracted using the RNeasy Mini Plus (Qiagen, Hilden, Germany). Complementary DNA was synthesized using the PrimeScript 1^st^ Strand Synthesis kit (Takara Bio, Japan). Quantitative PCR (qPCR) analysis was performed using the PowerUp^TM^ SYBR^TM^ Green mixture (Thermo Fisher Scientific) and the StepOnePlus Real Time PCR System (Thermo Fisher Scientific). Gene expression levels were calculated from Ct values, and the relationship between the *Ct* value and the logarithm of the copy number of a target gene was confirmed to be on a linear line using the corresponding isolated DNA and its serial dilutions as a standard. Thus, the gene expression levels of IL-8 and VEGF were normalized against that of β-actin in each sample. The following primers were used; β-actin, 5′-GCACAGAGCCTCGCCTT-3′ and 5′-GTTGTCGACGACGAGCG-3′; IL-8, 5′-CAAGAGCCAGGAAGAAACCA-3′ and 5′-AGCACTCCTTGGCAAAACTG-3′; VEGF, 5′-GGGCAGAATCATCACGAAGT-3′ and 5′-GCACACAGGATGGCTTGAAG-3′.

### Animal study

Balb *nu/nu* or C57BL/6J mice were purchased from Clea Japan Inc. (Tokyo, Japan). Mice were used for experiments at 2–3 months of age. For SW480 transplanted xenograft model, SW480 cells were subcutaneously implanted into male Balb *nu/nu* mice. The sample size was estimated from previous studies in tumor-bearing mice [8]. After 14 days, the size-matched tumor-bearing mice are randomly divided to four groups, and received divalent peptide3A5 with/without bevacizumab (Chugai Pharmaceutical, Tokyo, Japan) or 5-FU (Sigma-Aldrich. St. Louis, MO, USA) every 3 days. For generation of the Lewis lung cancer implantation mouse model, LLC cells were subcutaneously implanted into male C57BL/6J mice. After 10 days, the size-matched tumor-bearing mice are randomly divided to four groups, and received divalent peptide3A5 (10 mg/kg, i.p.) every 3 days. Anti-PD-1 antibodies (Biolegend, San Diego, CA, USA) were used for combination therapy. To determine the tumor volume, the longitudinal diameter (length) and transverse diameter (width) were determined. Tumor volume based on measurements by Vernier calipers was calculated as tumor volume = ½ (length × width^2^). For in vivo S100A8 stimulation, C57BL/6J mice were intravenously injected with mammalian-derived S100A8. Four hours after the *i.v*. injection, mice were sacrificed, and minced mouse lungs were digested with collagenase/dispase/DNase solution. Collected cells were incubated with anti-CD11b-PE (BD Bioscience), anti-Ly6G-FITC (BD Bioscience), and anti-Ly6C-APC (BD Bioscience) antibodies, and analyzed by a flow cytometry (Cytomics FC500; Beckman Coulter, Brea, CA, USA). For lung dissemination experiments, mice were pretreated for 1 h with divalent peptide3A5 (10 mg/kg, i.p.) prior to the last injection of tumor conditioned medium (TCM). After 24 h, PKH26 (Sigma-Aldrich)-fluorescent-labeled LLC cells were intravenously injected into TCM-stimulated C57BL6J mice. The mice were sacrificed after 16 h, and minced mouse lungs were digested with collagenase/dispase/DNase solution. The recruitment of MDSCs or PKH-26 labeled tumor cells in the lungs was determined by flow cytometry (CytoFlex; Beckman Coulter). All procedures performed on the mice were approved by the Animal Research Committee of Tokyo Women’s Medical University.

### Immunostaining

To immunostain tumor vasculatures, the cryo sections (6 µm) obtained from frozen primary tumors, were incubated with anti-CD31 (BD Pharmingen, Cat No. 550274), and/or anti-α-SMA-Cy3 (Sigma-Aldrich, Cat No. C6198) followed by AlexaFlour 488-conjugated rat IgG secondary antibodies (Thermo Fisher Scientific) were used to visualize the signals by a confocal microscopy (LSM-710; Carl Zeiss). DAPI staining was used for nuclear staining. For immunohistochemistry, the paraffin-embedded sections were cut 4 µm thick and then deparaffinized, rehydrated and then treated for antigen retrieval. The sections were incubated with the indicated antibody overnight, followed by incubation with ImmPRESS HRP Reagent (Vector, Newark, CA). ImmPACT DAB (Vector) was used for visualization of the signals. The images were captured by BZ-X800 (Keyence, Osaka, Japan).

### Statistics

Data are shown as the mean ± SD. Statistical significance analysis was completed by the two-sided Student’s *t* test, or Mann–Whitney *U* test (GraphPad Prism 6.0). *P* values <0.05 were considered statistically significant.

## Results

### S100A8 could be a therapeutic target against tumor progression

**Fig. 1:**
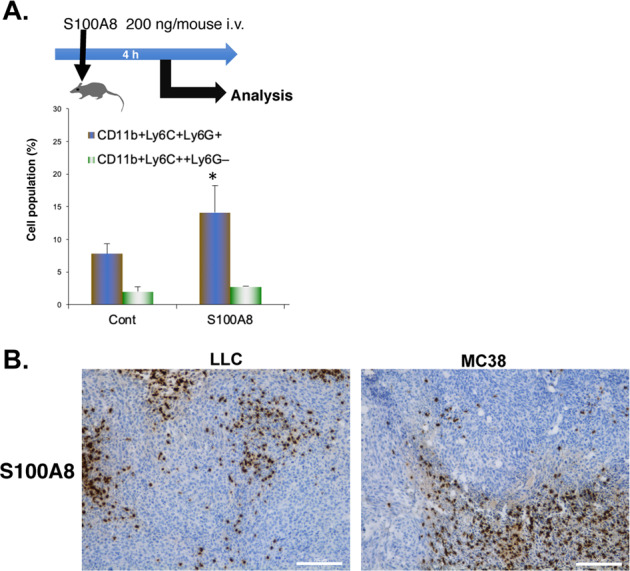
S100A8 could be a therapeutic target against tumor progression. **A** Administration of recombinant S100A8 protein induces pulmonary recruitment of MDSCs from bone marrow. Data are shown as mean ± SD. *P < 0.05 compared with control (Cont). **B** Immunohistochemical analysis of S100A8 in subcutaneous LLC or MC38 tumors in C57BL6 mice. Scale bar: 200 µm.

We previously showed that S100A8 is an endogenous TLR4 ligand, and that an anti-S100A8 neutralizing antibody can suppress tumor progression in tumor-bearing mice [4, 8]. Initially, we proposed that S100A8 induced in the lungs by the primary tumor contributed to pulmonary premetastatic niche formation. So far, S100A8/S100A9 has been described as a crucial factor for recruitment of MDSCs and immunosuppression in the tumor microenvironment [26]. To further investigate the effect of S100A8, mice were administered with S100A8 recombinant protein. Administration of S100A8 in mice increased the recruitment of MDSCs from the bone marrow to the lungs (Fig. 1A). In the tumor microenvironment, not only MDSCs, but also tumor cells can express TLR4 [8, 27, 28]. In tumor-bearing mice, S100A8 was expressed in infiltrating monocytes at the tumor surrounding area as well as found in intratumoral areas (Fig. 1B). As clinical relevance on S100A8, high levels of S100A8 expression are shown to be associated with poor prognosis in colorectal carcinoma or renal carcinoma (https://www.proteinatlas.org) [29]. In addition, Pan et al. [30] reported that the protein expression of S100A8 was significantly higher in cholangiocarcinoma than adjacent normal tissues, and that expression of S100A8 was associated with poor prognosis in aggressive cholangiocarcinoma. It has been reported that the relatively high expression of S100A8 is found in infiltrating monocytes in human colorectal cancer specimens [29]. Moreover, MDSCs infiltrate in primary metastasizing tumor sites when compared to non-metastasizing tumors [31]. These findings suggest that S100A8 could be a therapeutic target against tumor progression.

### The carboxyl-terminal region of human S100A8 binds to TLR4/MD-2 complex

**Fig. 2:**
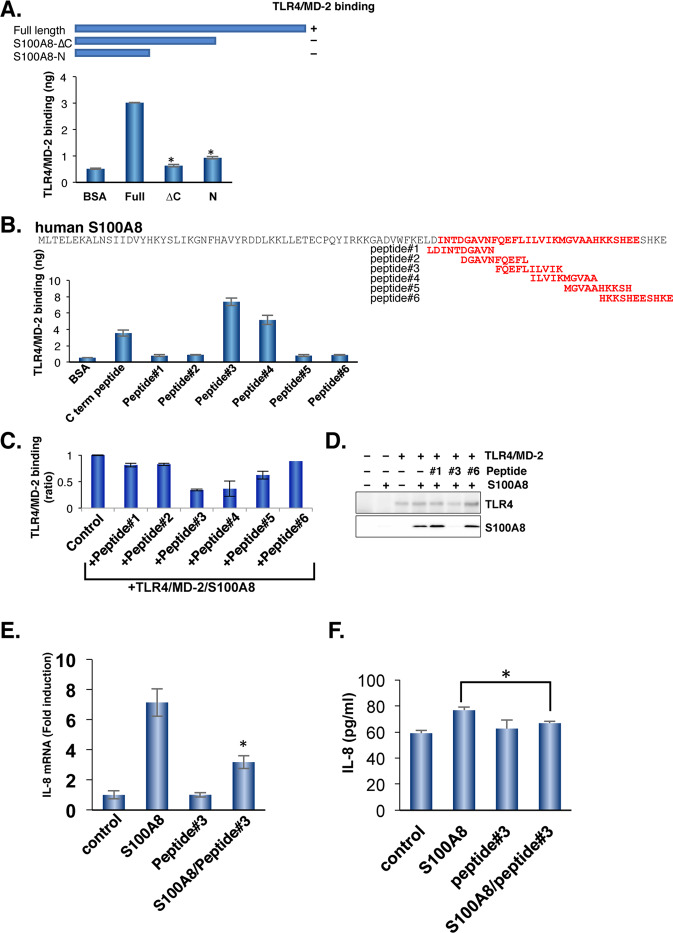
Identification of S100A8 binding sites to human TLR4/MD-2. **A** The human S100A8 binds to human TLR4/MD-2 complex. TLR4/MD-2 recombinant proteins were immobilized to the ELISA plate. After blocking, a full length or deletion mutant of S100A8 was added to the plate. The more detailed methods are described in Materials and Methods. Data are shown as mean ± SD. *P < 0.05 compared with full length of S100A8. **B** A series of overlapping S100A8 synthetic peptides. The TLR4/MD-2 recombinant proteins were added to each well of peptide immobilized plates. The affinity to TLR4/MD-2 was determined. Data are shown as mean ± SD. **C** The competitive activity was determined using ELISA. Data are shown as mean ± SD. **D** The TLR4/MD-2 was mixed with S100A8 with/without the indicated peptides, and immunoprecipitated using an anti-TLR4 antibody. TLR4 or S100A8 was visualized. Similar results were obtained in three independent experiments. **E** Peptide #3 reduces the expression of S100A8-mediated IL-8 induction. Total RNAs from SW480 cells with/without treatment of the peptide candidates were extracted and qPCR analysis was performed using relevant primer sets and SYBR green. Relative expression levels were compared. Data are shown as mean ± SD. *P < 0.05 compared with treatment of S100A8. **F** Peptide #3 suppresses S100A8-mediated IL-8 secretion in SW480 cells. Data are shown as mean ± SD. *P < 0.05 compared with treatment of S100A8.

We have previously shown that mouse S100A8 binds to the mouse TLR4/MD-2 complex via the carboxyl-terminal region of S100A8 [8]. To obtain a specific inhibitor of human S100A8 against the human TLR4/MD-2 complex, we investigated the human S100A8 binding sites against the human TLR4/MD-2 complex using a strategy similar to that used with mouse S100A8. Alignment of human S100A8 with mouse S100A8 showed 55.9% identity, suggesting that the carboxyl-terminal region of S100A8 could be responsible for TLR4/MD-2 binding. Similar to the finding using mouse S100A8, the deletion of the carboxyl-terminal region of human S100A8 failed to bind to human TLR4/MD-2 (Fig. 2A). To further narrow down the binding site against the TLR4/MD-2 complex, five overlapping peptides were generated, and the ability of TLR4/MD-2 binding was tested. Peptide #3 most strongly bound to the TLR4/MD-2 complex among a series of S100A8-derived peptides within the carboxyl terminal region (Fig. 2B). Peptide #3 also competed for binding between S100A8 and the TLR4/MD-2 complex, indicating that the amino acid sequence of peptide #3 contains the binding sites for the TLR4/MD-2 complex (Fig. 2C, D). Of note, peptide #3 shared half of the amino acid sequence with peptide #4.

### Peptide #3 inhibits S100A8-mediated interleukin-8 and VEGF production and suppresses cell migration in SW480 cells

To test the effect of peptide #3 in situ, we chose SW480 cells that expressed endogenous TLR4/MD-2 and efficiently responded to S100A8. As shown in Fig. 2E, S100A8-mediated TLR4/MD-2 activation induced IL-8 production in SW480 cells, similar to that by other TLR4 ligands [32]. Importantly, peptide #3 alone could bind to TLR4/MD-2, but failed to induce IL-8 production, indicating that peptide #3 can function as a competitive antagonist against TLR4/MD-2. Furthermore, S100A8-mediated IL-8 secretions were also suppressed by pretreatment with peptide#3 (Fig. 2F). *VEGF* is another NFκB-mediated responsible gene. Peptide #3 suppressed S100A8-induced VEGF induction (Supplementary Fig. 1A). We previously showed that S100A8 induces cell migration in a TLR4-dependent manner [8]. S100A8-mediated cell migration was significantly inhibited by treatment with peptide #3 (Supplementary Fig. 1B). These results indicate that peptide #3 can function as a S100A8 competitive inhibitory peptide, but cannot perform agonistic activities for TLR4/MD-2.

### S100A8-Lys77Ala mutant significantly reduces the capacity of both TLR4/MD-2 binding and IL-8 induction

**Fig. 3:**
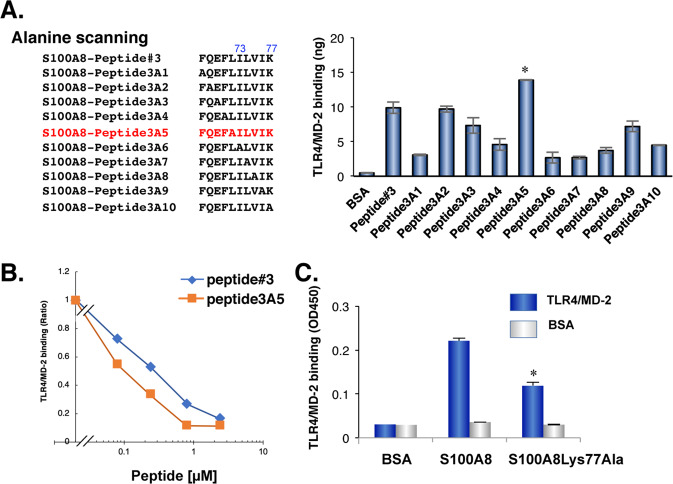
Identification of high affinity peptides to TLR4/MD-2 using alanine scanning based on the peptide #3 amino acid sequences. **A** Lists of peptides used for alanine scanning. Peptide3A5 displayed increased binding affinity to TLR4/MD-2 when compared to that of parental peptide#3. *P = 0.013. **B** Peptide3A5 is more potent for competitive inhibition on S100A8-TLR4/MD-2 complex than peptide #3. Similar results were obtained in three independent experiments. **C** The S100A8Lys77Ala recombinant protein decreased affinity to TLR4/MD-2. Data are shown as mean ± SD. *P < 0.05 compared with of full length of wild type (WT) S100A8.

To further identify the site(s) responsible for binding, we performed alanine scanning using the sequence of peptide #3. The substitution of Ile73, Leu74, Val75, or Lys77 with alanine diminished the S100A8 activity of TLR4/MD-2 binding (Fig. 3A). In contrast, the substitution of Leu72 with alanine (peptide3A5) increased the affinity for TLR4/MD-2 (Fig. 3A). Importantly, peptide3A5 competed with the S100A8-TLR4/MD-2 complex at a lower dose than peptide #3, indicating that peptide3A5 was a more potent inhibitor of S100A8 than peptide #3 (Fig. 3B).

We then generated the S100A8-Lys77Ala recombinant protein. The S100A8-Lys77Ala mutant hardly bound to the TLR4/MD-2 complex (Fig. 3C), and had no effect on IL-8 production in SW480 cells (data not shown). Taken together, these results suggest that Lys77 of S100A8 could be one of the sites responsible for TLR4/MD-2 binding, and that peptide3A5 would provide the competitive inhibitory activity against S100A8-TLR4/MD-2 complex.

### Divalent peptide3A5 is a first candidate of S100A8 inhibitory peptides

**Fig. 4:**
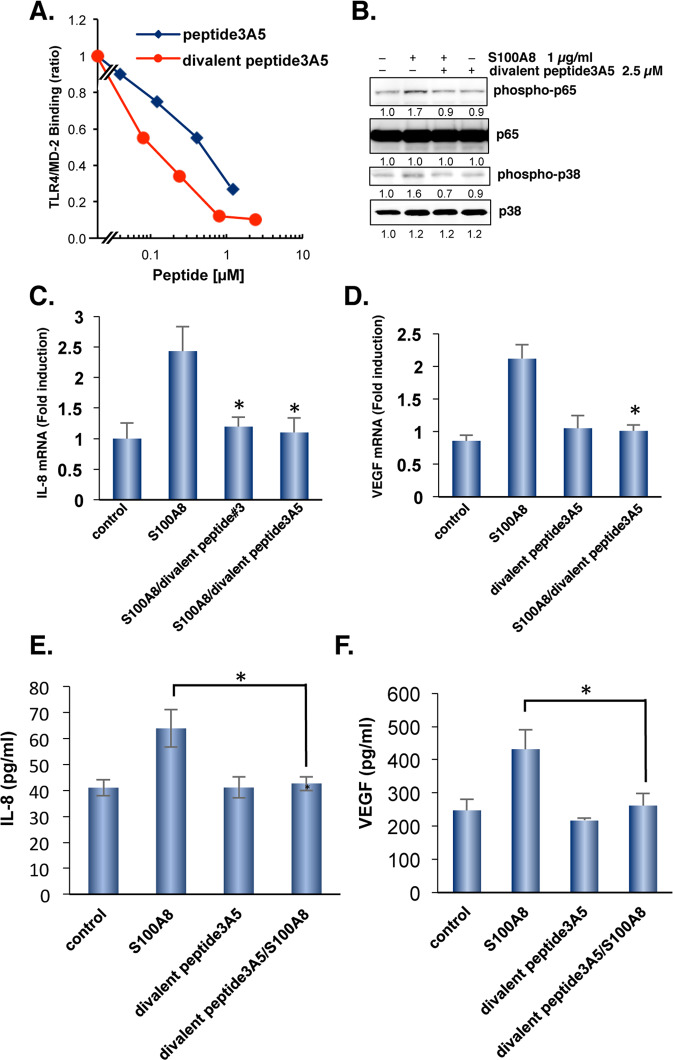
Improvement of competitive inhibition activity by divalent peptide3A5. **A** The binding capacity was measured at the indicated concentration using an ELISA-based assay. Similar results were obtained in three independent experiments. **B** SW480 cells were pretreated with 2.5 µM of divalent peptide3A5, and then stimulated with human S100A8 recombinant protein. After 30 min, cell lysates were subjected to western blotting using the indicated antibody. The divalent peptide#3 or peptide3A5 reduced the expression of S100A8-mediated IL-8 (**C**) and VEGF (**D**) induction. Total RNAs from SW480 cells with/without treatment of the peptide candidates were extracted and qPCR analysis was performed using relevant primer sets and SYBR green. Relative expression levels were compared. Peptide #3 suppresses S100A8-mediated IL-8(**E**) and VEGF (**F**) secretion in SW480 cells. Data are shown as mean ± SD. *P < 0.05 compared with treatment of S100A8.

TLR4/MD-2 dimerizes upon ligand binding. To attempt the inhibitory peptide fitted into dimerized forms, we generated a divalent peptide of peptide3A5 using Fmoc-Lys (Fmoc)-OH to form two branches in the peptide chain. Competitive inhibition with divalent peptide3A5 was observed at a lower dose range than the effect of the peptide3A5 monomer (Fig. 4A). We then examined the effect of divalent peptide3A5 on TLR4/MD-2 downstream signaling. Upon TLR4 activation by S100A8, S100A8 induced phosphorylation of p38 MAP kinase, followed by activation of NF-κB signaling, as previously reported [8]. Pretreatment with divalent peptide3A5 inhibited S100A8-induced phosphorylation of both p38 and p65 (NF-κB) by 1.3- and 1.7-fold, respectively (Fig. 4B). Divalent peptide3A5 efficiently inhibited S100A8-mediated IL-8 or VEGF induction (Fig. 4C, D). As consistent with the effect on IL8 or VEGF mRNA induction, divalent peptide3A5 suppressed S100A8-mediated induction of IL-8 and VEGF (Fig. 4E, F).

**Fig. 5:**
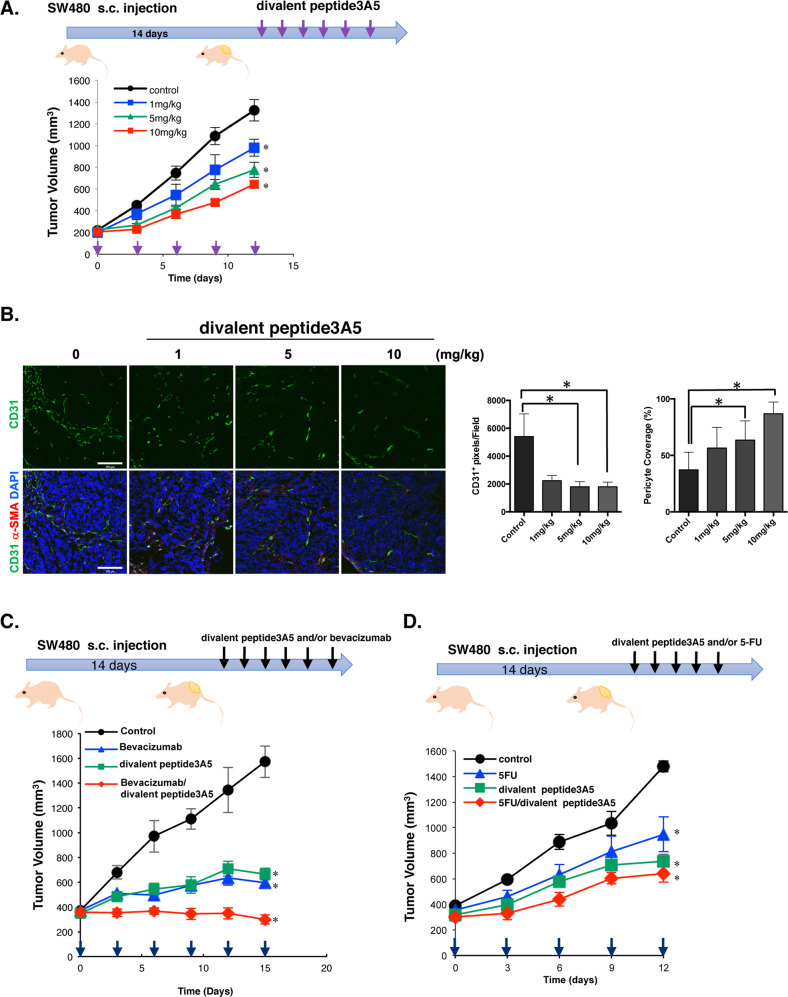
Divalent peptide3A5 inhibits tumor growth in SW480-tranplanted xenograft models. **A** Schematic representation of the experiment. Briefly, SW480 cells were implanted s.c. into Balb *nu/nu* mice (*n* = 5–6 mice per group). Fourteen days later, tumor-bearing mice started to receive divalent peptide3A5 peptide at various concentrations every 3 days. Tumor volumes for individual mice were determined by caliper measurements at the indicated time points. Data are shown as mean ± SEM. *P < 0.05 compared with Control. **B** The tumor vasculatures were visualized using anti-CD31 and anti-α-SMA antibodies. The representative photos are shown. Scale bar: 200 µm. Pericyte coverage was calculated the signal of α-SMA divided with the signal of CD31. Data are shown as mean ± SD. *P < 0.05 compared with control tumor-bearing mice. **C** Using combination therapy, divalent peptide3A5 synergistically inhibited tumor growth with bevacizumab in SW480-transplanted xenograft models. Divalent peptide3A5 (5 mg/kg, i.p) and/or bevacizumab (4 mg/kg, i.p) were administered every 3 days. Tumor volumes for individual mice were determined at the indicated time points. Data are shown as mean ± SEM. *P < 0.05 compared with Control. **D** Divalent peptide3A5 synergistically inhibits tumor growth with 5-FU combination in SW480-transplanted xenograft models. Divalent peptide3A5 (5 mg/kg, i.p) and/or 5-FU (20 mg/kg, i.p) were administrated every 3 days. Tumor volumes for individual mice were determined at the indicated time points.

To evaluate the toxicity of divalent peptide3A5 at 10 mg/kg, we performed the biochemical test, and found that serum levels of AST, ALT, LDH displayed as normal (Supplementary Table 1). In addition, there is no significant difference in body weight and hematological test with treatment of divalent peptide3A5 (Supplementary Table 1). We then examined the effect of divalent peptide3A5 on tumor growth in vivo. It dose-dependently inhibited tumor growth in the SW480-transplanted xenograft model (Fig. 5A). The divalent peptide3A5 suppressed tumor cell proliferation and caused caspase-3 activation in subcutaneous SW480 tumors of Balb *nu/nu* mice (Supplementary Fig. 2). In addition, the tumor vasculature significantly diminished with the treatment of divalent peptide3A5 in a dose-dependent manner (Fig. 5B). Notably, divalent peptide3A5 significantly increased the pericyte coverage at the dosage of 5 mg/kg or 10 mg/kg, suggesting that divalent peptide3A5 induce tumor vascular normalization (Fig. 5B).

### Divalent peptide3A5 synergistically enhances Bevacizumab-induced anti-tumoral activity

Because divalent peptide3A5 alone inhibited tumor growth in vivo through suppression of tumor angiogenesis (Fig. 5B), we next evaluated the effect of the divalent peptide3A5 when used in combination with bevacizumab post-chemotherapy. The tumor growth reduced by 81 % bevacizumab (4 mg/kg) alone compared to 106% using combination therapy, suggesting that combination therapy with divalent peptide3A5 synergistically inhibited tumor growth in SW480-xenograft models (Fig. 5C). Similarly, but to a lesser extent, 5-FU combination therapy with divalent peptide3A5 synergistically suppressed tumor growth in SW480-xenograft models (Fig. 5C).

### Effect of divalent peptide3A5 in syngeneic tumor-transplanted models

**Fig. 6:**
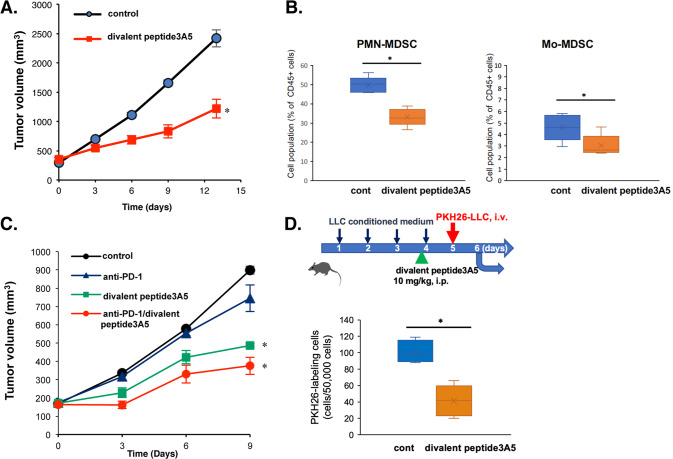
Divalent peptide3A5 suppresses tumor progression in an LLC transplanted-syngeneic mouse model. **A** Monotherapy of divalent peptide3A5 (10 mg/kg, i.p). Briefly, LLC cells were implanted s.c. into C57BL/6 J mice. Ten days later, tumor-bearing mice were administered divalent peptide3A5 every 3 days. Tumor volumes for individual mice were determined at the indicated time points. Each group included five mice. Data are shown as mean ± SEM. *P < 0.05 compared with Control. **B** The treatment of divalent peptide3A5 reduces cell number of MDSCs in peripheral blood of LLC-tumor-bearing mice. Data are shown as mean ± SD. *P < 0.05 compared with Control. **C** Combination therapy with anti-PD-1 antibody in LLC-tumor-bearing mice. Divalent peptide3A5 with/without anti-PD-1 antibody was administered every 3 days. Tumor size was determined at the indicated time points. Data are shown as mean ± SEM. Each group included 5–6 mice. *P < 0.05 compared with Control. **D** Divalent peptide3A5 suppresses the pulmonary recruitment of PKH26-labeled LLC cells in TCM-sensitized mice. Each group included 5–6 mice. The mice were administered tumor conditioned medium once daily for five consecutive days. Divalent peptide3A5 (10 mg/kg, i.p) was pre-administered 1 h before the last injection. The PKH-26 labeled LLC tumor cells were injected intravenously, the mice were sacrificed, and the cell number was analyzed after 16 h. Each group included 5–6 mice. Data are shown as mean ± SD. *P < 0.05 compared with Control.

Because S100A8 inhibitory peptides target S100A8-TLR4/MD-2, which plays a role in innate immunity, the complete functional activities of S100A8 inhibitory peptides are expected to be observed in immunocompetent models. To date, syngeneic models are one of the only options to test physiological interactions between tumors and immune cells. As similar to the effect in Balb *nu/nu* mice, the divalent peptide3A5 did not display any toxicity in C57BL6 mice (Supplementary Table 1). As shown in Fig. 6A, the administration of divalent peptide3A5 every three days inhibited in vivo tumor growth in LLC tumor-bearing mice. Importantly, treatment with divalent peptide3A5 reduced the cell population of MDSCs (Fig. 6B). We then examined the combination therapy with anti-programmed death (PD)-1 antibody in LLC-tumor-bearing mice. LLC cells were identified as non-responders to anti-PD-1/programmed death-ligand 1 (PD-L1) therapy. Combination therapy with divalent peptide3A5 improved the efficacy of anti-PD-1 (Fig. 6C). Of note, the cell numbers of intratumoral infiltrating CD8^+^ cells increased with the treatment of divalent peptide3A5 alone, and the combination therapy with anti-PD-1 antibody (Supplementary Fig. 3A). We then examined the effect of divalent peptide3A5 in mouse MC38 colorectal cancer cells used as anti-PD-1 responder cells. We previously confirmed that MC38 endogenously expressed TLR4/MD-2 [8]. The administration of divalent peptide3A5 with anti-PD-1 antibody significantly improved the efficacy of anti-PD-1 therapy for tumor suppression (Supplementary Fig. 3B).

### Divalent peptide3A5 inhibits the MDSC recruitment and pulmonary premetastatic niche formation

As shown in Fig. 6A, C, treatment with divalent peptide3A5 suppressed tumor growth in tumor-bearing mice. To address the effect of divalent peptide3A5 in premetastatic niche formation more directly, mice were treated with tumor-conditioned medium [4] at the indicated time points, followed by injection of fluorescent PKH26-labeled tumor cells (Fig. 6D). Pretreatment with peptide3A5 suppressed the pulmonary recruitment of tumor cells (Fig. 6D), indicating that divalent peptide3A5 can inhibit the formation of a premetastatic niche and metastasis. These results suggest that divalent peptide3A5 can inhibit not only formation of tumor microenvironment, also premetastatic niche formation.

### Peptide3A5-derived divalent peptide screening

**Fig. 7:**
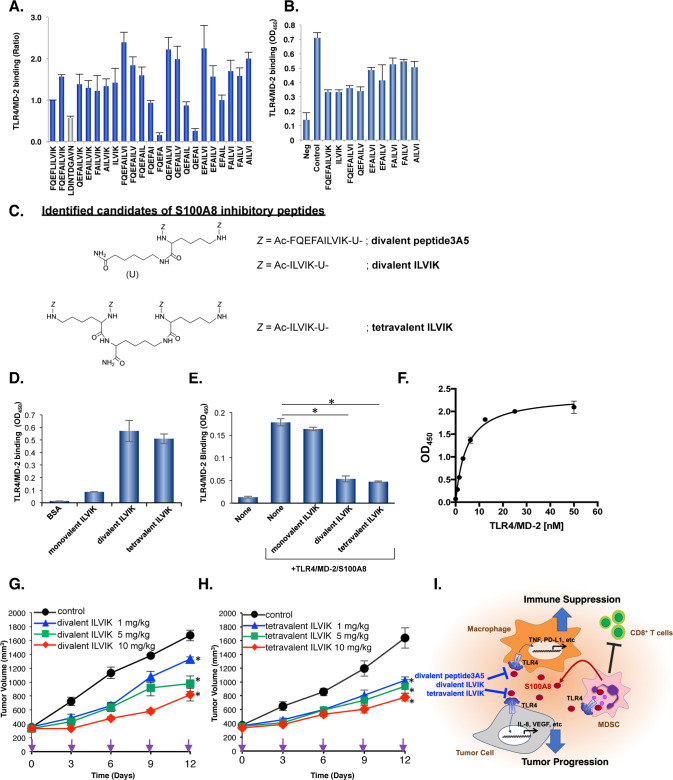
Isolation of multivalent S100A8 inhibitory peptides using divalent peptide libraries based on the sequences of peptide3A5. **A** Divalent peptide library screening based on peptide3A5 sequences. The binding affinities to TLR4/MD-2 complex were determined using an ELISA-based assay. Data are shown as mean ± SD. **B** Eight candidates were tested using a competitive inhibitory assay. **C** The structures of the identified S100A8 inhibitory peptides are shown. Amino hexanoic acid (U) was used as a spacer. **D** The binding affinities of ILVIK derivatives to TLR4/MD-2 complex. **E** Monovalent, divalent, tetravalent, ILVIK peptides were tested using a competitive inhibitory assay. Data are shown as mean ± SD. *P < 0.05 compared with TLR4/MD-2/S100A8. **F** TLR4/MD-2 binding to immobilized tetravalent ILVIK. After blocking for nonspecific binding, the TLR4/MD-2 proteins were added with various concentrations. One representative out of three independent experiment is shown. **G** Divalent ILVIK peptide inhibits tumor growth in SW480-transplanted xenograft models. Tumor volumes for individual mice were determined at the indicated time points. Divalent ILVIK peptide was administered at various concentrations every 3 days. Data are shown as mean ± SEM. *P < 0.05 compared with Control. **H** Tetravalent ILVIK peptide inhibits tumor growth in SW480-transplanted xenograft models. Tumor volumes for individual mice were determined at the indicated time points. Tetravalent peptide was administered at various concentrations every 3 days. Data are shown as mean ± SEM. *P < 0.05 compared with Control. **I** The possible mechanism(s) which involved in multivalent S100A8 inhibitory peptides. In this study, we developed divalent peptide3A5, divalent ILVIK, and tetravalent ILVIK, which are S100A8 competitive inhibitors against TLR4/MD-2 complex. These peptides suppress the induction of cytokines/chemokines in both tumor cells and macrophages, which would involve in anti-tumor activity by multivalent S100A8 inhibitory peptides.

To further develop S100A8 inhibitor by narrowing down amino acid sequences of peptide3A5 for TLR4/MD-2 binding, we performed peptide screening using a series of peptide3A5-derived divalent peptide libraries. Intriguingly, the FQEFA dimer, which deleted the last five amino acids, diminished the capacity of binding to TLR4/MD-2 (Fig. 7A). In contrast, the deletion mutant of the FQEFA amino acid sequences sustained the competitive inhibitory activity. The divalent ILVIK was a candidate inhibitor among a series of petide3A5 derivatives (Fig. 7B).

### Divalent and tetravalent ILVIK, but not ILVIK monomer are potent S100A8-TLR4/MD-2 competitive inhibitors

Modification of multivalency is often known to produce clustering effects against target molecules. We previously developed a novel tetravalent peptide-based inhibitor against Shiga toxin using multivalent peptide library screening [23]. We then synthesized a tetravalent peptide with four ILVIK motifs. To synthesize the divalent or tetravalent peptide, a monolysine or a polylysine core was utilized, respectively, to bifurcate at each end, as previously described [23]. The N-terminus of all three peptides was acetylated to prevent proteolytic degradation. The structure of S100A8 inhibitory peptides are shown in Fig. 7C. Both competitive inhibitory activity and binding affinity to TLR4/MD-2 by tetravalent ILVIK were similar to those of the divalent ILVIK peptide, but notably, the monomeric ILVIK peptide did not perform these activities (Fig. 7D, E). These results suggested that the multivalent approach with ILVIK was advantageous as a S100A8 competitive inhibitor against the TLR4/MD-2 complex. The Kd value of tetravalent ILVIK to the TLR4/MD-2 complex was calculated as 4.58–8.78 nM (Fig. 7F). Similar to divalent peptide3A5, divalent ILVIK competitively inhibited S100A8 binding to TLR4/MD-2 and inhibited tumor growth in subcutaneously transplanted SW480 cells in a dose-dependent manner (Fig. 7G). When compared to the divalent ILVIK peptide, tetravalent ILVIK peptide more effectively inhibited tumor progression in a SW480-transplanted xenograft model (Fig. 7H). Therefore, these results suggested that the multivalent technique of targeting amino acid sequences could enhance the anti-tumoral activities by increasing affinity against TLR4/MD-2 (Fig. 7I).

## Discussion

We previously showed that treatment with anti-S100A8/S100A9 antibodies inhibited metastasis in mouse lung carcinoma (3LL)-bearing mice [4]. More recently, we found that Eritoran inhibited tumor growth through immunomodulation and vascular normalization, and that the carboxyl-terminal sequences of mouse S100A8 were necessary for binding to the mouse TLR4/MD-2 complex using a binding assay and CyClus docking simulation [8].

Unlike other TLRs, TLR4 can activate two major downstream pathways: myeloid differentiation factor 88 (MyD88)- and TIR domain-containing adaptor protein-inducing interferon β (TRIF)-dependent pathways. S100A8 is known to be an endogenous ligand of TLR4, but preferentially activates the MyD88-dependent pathway, which causes the induction of inflammatory cytokines, such as IL-8, IL-6, and TNF. Thus, an S100A8 specific inhibitor does not affect TRIF-mediated signaling to induce interferons. In this study, we focused on S100A8 as a therapeutic target against aggressive cancers in an effort to improve tumor-immune responses. We have developed divalent peptide3A5 (FQEFAILVIK), divalent ILVIK, and tetravalent ILVIK peptide as novel multivalent S100A8-TLR4/MD-2 competitive inhibitors.

The sequence identity between mouse S100A8 and human S100A8 is approximately 55.9%; however, Ca^2+^ binding sites are highly conserved in both species. Based on alanine scanning of monomeric peptide#3 (FQEFLILVIK), we found that the last amino acid of peptide#3 (Lys77) is one of the sites responsible for TLR4/MD-2 binding (Fig. 3C). Surprisingly, the substitution of alanine on the fifth amino acid of peptide#3 increased the affinity for TLR4/MD-2 (Fig. 3A). This serendipity allowed us to generate competitive inhibitors based on the amino acid sequence of peptide3A5 (FQEFAILVIK). In addition, we performed a divalent peptide library approach using peptide3A5 to narrow down the length of the peptides. As shown in Fig. 7A, deletion of the second half lost the binding ability to TLR4/MD-2, whereas the divalent peptide of the second half sustained binding capability to TLR4/MD-2, with competitive activities (Fig. 7B). It should be noted that monomeric ILVIK failed to obtain the competitive inhibitory activities against the TLR4/MD-2 complex (Fig. 7E), suggesting that a multivalent approach is necessary to obtain competitive inhibitory activity of ILVIK peptide (Fig. 7E).

Combination therapy using divalent peptide3A5 and bevacizumab synergistically was found to be suppressed tumor progression in SW480 xenograft models (Fig. 5C), and that divalent peptide3A5 increased the efficacy of anti-PD-1 therapy in two syngeneic models (Fig. 6C and Supplementary Fig. 3B). We also found that cell number of intratumor infiltrating CD8^+^ cells increased with the treatment of divalent peptide3A5 alone, and the combination therapy with anti-PD-1 antibody (Supplementary Fig. 3A). It has been reported that S100A8 upregulated PD-L1 in macrophages via a TLR4-dependent pathway [29], and we previously found that the inhibition of TLR4 by Eritoran increased intratumoral infiltration of CD8^+^ cells in LLC-tumor-bearing mice [8]. Therefore, we assume that divalent peptide3A5 would provide anti-tumor activity with similar mechanism to Eritoran, which may improve the efficacy of anti-PD-1 antibody, and that multivalent S100A8 inhibitory peptides can be useful to improve the efficacy of other chemotherapeutic agents.

To date, Eritoran and TAK-242 (Resatorvid) are known to be TLR4 inhibitors [33, 34]. The difference between TAK-242 and our inhibitory peptides is the target molecules. Our S100A8 inhibitory peptides target the site responsible for TLR4/MD-2 at cell surface (Fig. 7I), whereas TAK-242 inhibits TLR4 at intracellular domains. Qin et al. previously generated two peptibodies, peptide-Fc fusion proteins, targeting mouse MDSCs using a competitive peptide phage display platform [35]. Peptibodies recognize the cell surface of mouse MDSCs. Although peptibodies also recognize S100A9, mouse MDSCs are distinguished by other cell surface markers that are quite different from those in humans, as described in the “Introduction” section. As we found that mobilization of MDSCs is induced by S100A8, which is a TLR4 endogenous ligand, we used a different strategy to isolate candidate peptides of S100A8 inhibitors.

Initially, we thought that S100A8 mainly plays a role in premetastatic niche formation, but several studies have reported that various types of cancer cells also express TLR4 at the cell surface, and that its expression is associated with poor prognosis in breast cancer and other types of cancer [36, 37]. Therefore, S100A8-TLR4/MD-2 may play a crucial role in both the host cells and primary tumors. Accordingly, the S100A8-TLR4/MD-2 axis could be a novel therapeutic target against cancer progression and metastasis by immunomodulation and vascular normalization.

Moreover, Fleming et al. recently reported that tumor cell-derived extracellular vesicles convert normal myeloid cells into functional MDSCs by regulating the expression of PD-L1 via TLR4 signaling [38]. TLR4-induced TGF-β expression has also been associated with the transformation of fibroblasts into cancer-associated fibroblasts in the tumor microenvironment, facilitating cancer cell proliferation and tumor growth [39–41]. Therefore, targeting the premetastatic niche-promoting molecular components to prevent metastasis may be an attractive approach for cancer therapy.

Chemoresistance, recurrence, and metastasis are the major causes of cancer-related death. Elucidation of the underlying mechanism(s) could contribute to establishing suitable therapeutic targets. Notably, metastatic tumors tend to disseminate to distant organs. For example, aggressive colon cancers typically metastasize to the liver. We found that S100A8 plays an important role in premetastatic niche formation, and not only in the lungs specifically (Deguchi, unpublished data). Our results suggest that S100A8 universally induces the mobilization of MDSCs and other inflammatory monocytes from the bone marrow. As mentioned above, S100A8 is one of the major inflammatory cytokines involved in acute and chronic inflammation. Interestingly, it has been reported that S100A8 is upregulated in patients with severe COVID-19 [42]. Therefore, in addition to cancer, it is likely that the S100A8-TLR4/MD-2 axis is a promising therapeutic target against other inflammation-associated diseases.

In summary, we developed multivalent S100A8 competitive antagonists. As mentioned, S100A8 is known to be involved in multiple pathways; therefore, S100A8 inhibitory peptides could have potential pharmaceutical application, but this aspect requires further study to verify in various models.

## Supplementary information

SupplFig1

SupplFig2

SupplFig.3

SupplTable 1

## Data Availability

All study data are included in this article.
