# Supplementary figures and images for: Novel multivalent S100A8 inhibitory peptides attenuate tumor progression and metastasis by inhibiting the TLR4-dependent pathway
Source: Cancer Gene Ther. 2023 Mar 17;30(7):973–84. doi: 10.1038/s41417-023-00604-3 (PMC10021052; doi:10.1038/s41417-023-00604-3)

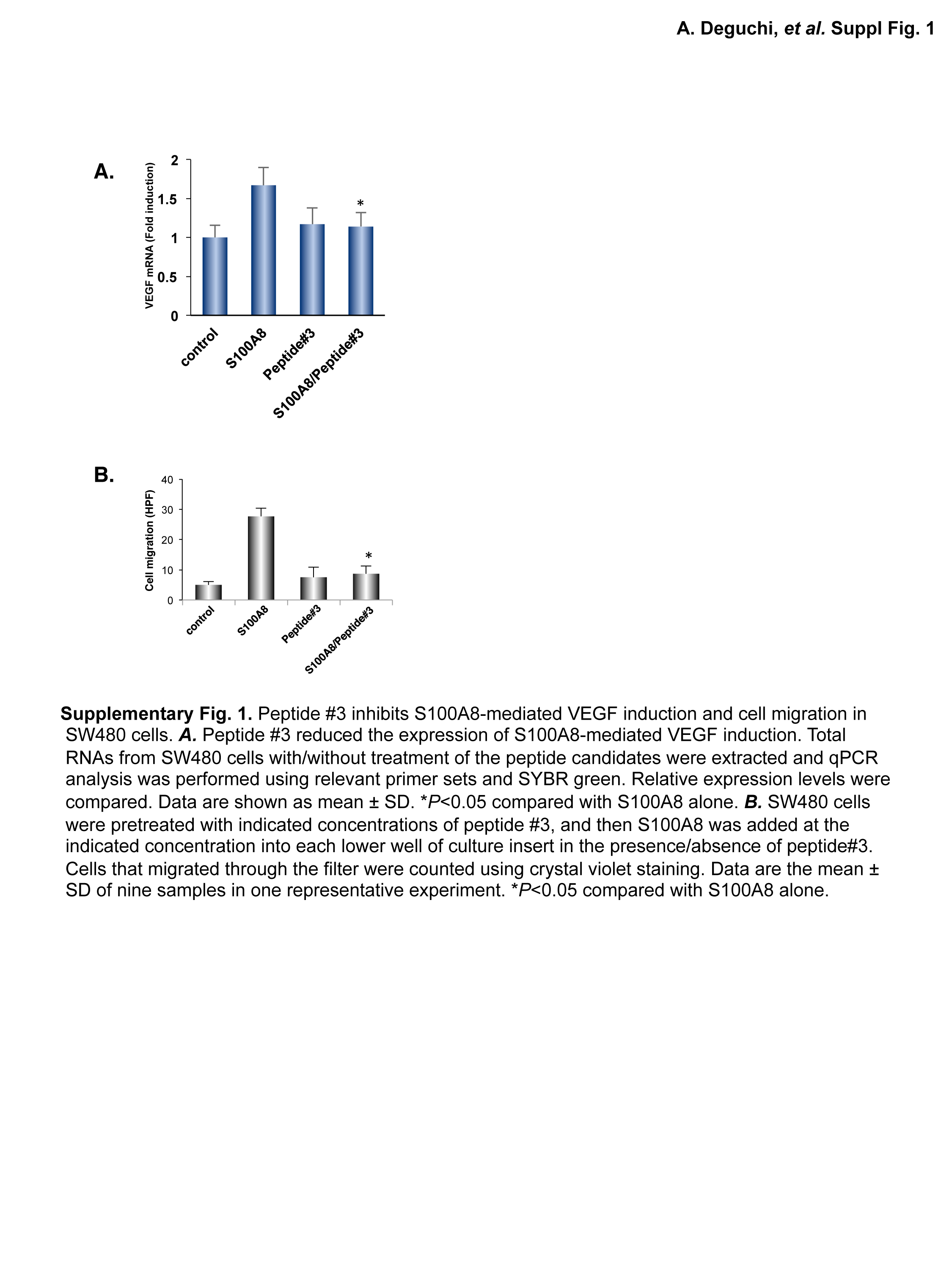

Supplement: Supplementary file 1 — SupplFig1 [file 41417_2023_604_MOESM1_ESM.tif]

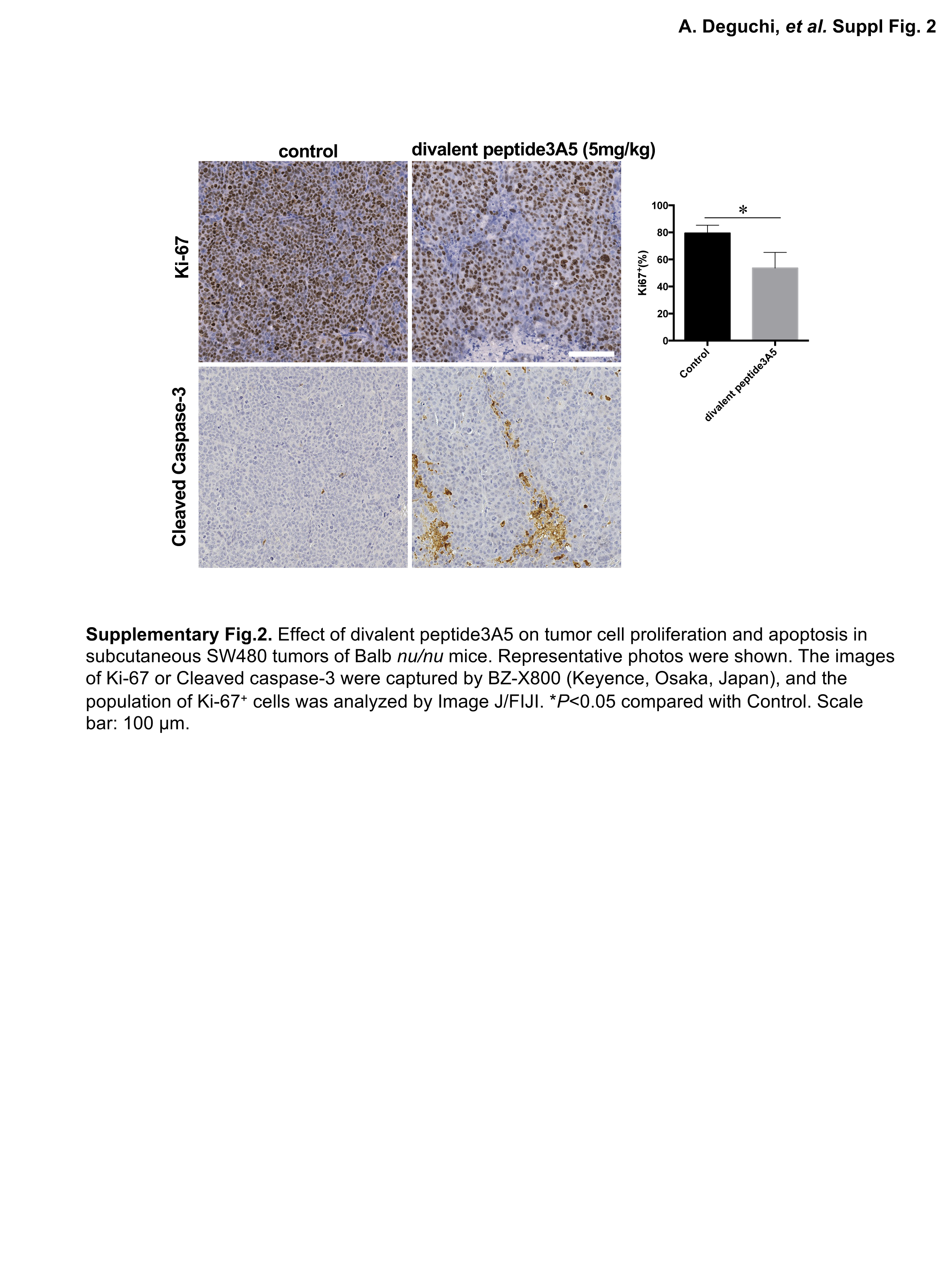

Supplement: Supplementary file 2 — SupplFig2 [file 41417_2023_604_MOESM2_ESM.tif]

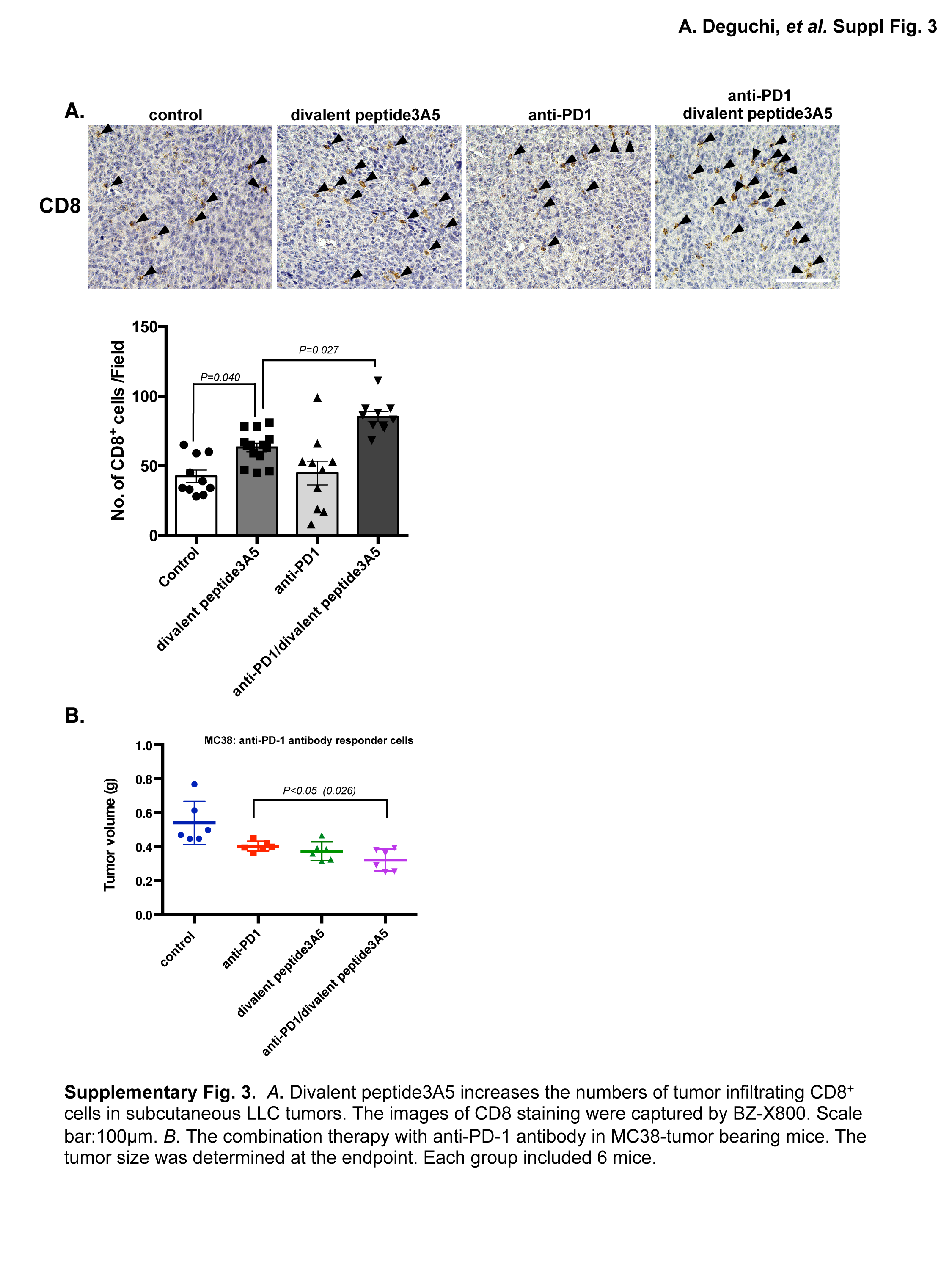

Supplement: Supplementary file 3 — SupplFig.3 [file 41417_2023_604_MOESM3_ESM.tif]

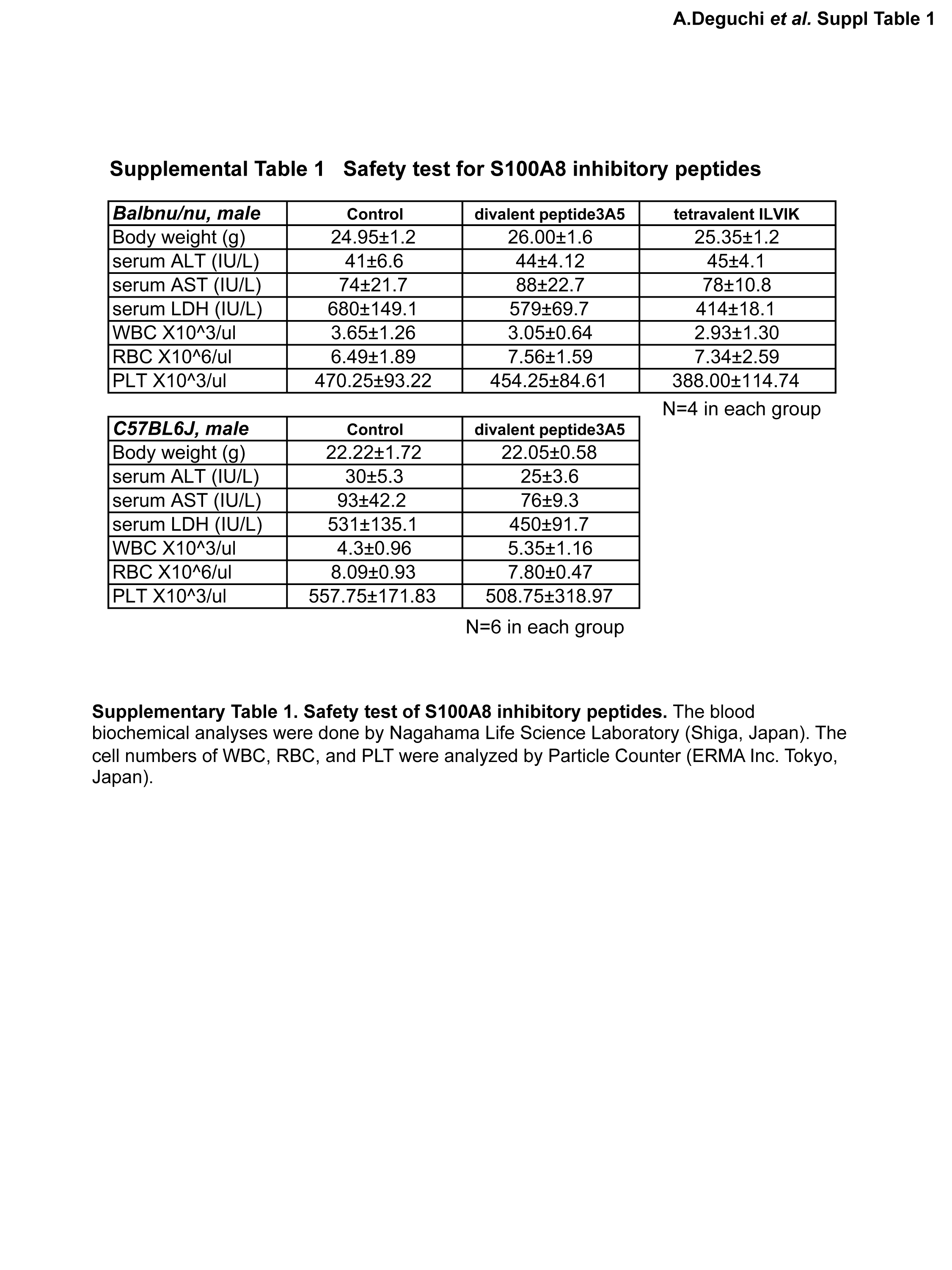

Supplement: Supplementary file 4 — SupplTable 1 [file 41417_2023_604_MOESM4_ESM.tif]
